# Enhanced Radiosensitization for Cancer Treatment with Gold Nanoparticles through Sonoporation

**DOI:** 10.3390/ijms21218370

**Published:** 2020-11-08

**Authors:** Shao-Lun Lu, Wei-Wen Liu, Jason Chia-Hsien Cheng, Lien-Chieh Lin, Churng-Ren Chris Wang, Pai-Chi Li

**Affiliations:** 1 Graduate Institute of Biomedical Electronics and Bioinformatics, National Taiwan University, Taipei 10617, Taiwan; b91401084@ntu.edu.tw (S.-L.L.); wwliu0801@gmail.com (W.-W.L.); jasoncheng@ntu.edu.tw (J.C.-H.C.); jacob821102@gmail.com (L.-C.L.); 2 Division of Radiation Oncology, National Taiwan University Hospital, Taipei 100229, Taiwan; 3 Graduate Institute of Oncology, National Taiwan University College of Medicine, Taipei 100229, Taiwan; 4 Department of Chemistry and Biochemistry, National Chung-Cheng University, Chia-Yi 621301, Taiwan; checrw@ccu.edu.tw; 5 Department of Electrical Engineering, National Taiwan University, Taipei 10617, Taiwan

**Keywords:** sonoporation, microbubbles, gold nanoparticles, cavitation, radiotherapy, radiosensitization

## Abstract

We demonstrate the megavoltage (MV) radiosensitization of a human liver cancer line by combining gold-nanoparticle-encapsulated microbubbles (AuMBs) with ultrasound. Microbubbles-mediated sonoporation was administered for 5 min, at 2 h prior to applying radiotherapy. The intracellular concentration of gold nanoparticles (AuNPs) increased with the inertial cavitation of AuMBs in a dose-dependent manner. A higher inertial cavitation dose was also associated with more DNA damage, higher levels of apoptosis markers, and inferior cell surviving fractions after MV X-ray irradiation. The dose-modifying ratio in a clonogenic assay was 1.56 ± 0.45 for a 10% surviving fraction. In a xenograft mouse model, combining vascular endothelial growth factor receptor 2 (VEGFR2)-targeted AuMBs with sonoporation significantly delayed tumor regrowth. A strategy involving the spatially and temporally controlled release of AuNPs followed by clinically utilized MV irradiation shows promising results that make it worthy of further translational investigations.

## 1. Background

Radiotherapy (RT), as a powerful anticancer modality, is applied to 40% of patients who are eventually cured [1]. However, RT is not a selective antitumor treatment, and so the main challenge is maximizing its therapeutic efficacy while minimizing damage to the surrounding healthy tissues. Radiosensitization is a process to increase the efficacy of RT by introducing radiosensitizers, which are molecules/materials with the ability to enhance the radiosensitivity of tumor cells [2,3]. Conventional radiosensitizers for clinical applications include alkylating agents (platinum, nitrogen mustards, etc.), nucleosides analogues (gemcitabine, cytarabine, etc.), antimetabolites (5-fluorouracil, methotrexate, etc.), taxanes (docetaxel, paclitaxel), and targeted molecules (bevacizumab, etc.) [4]. Most of the clinically utilized sensitizers are administered systemically, and so generalized adverse effects remain of great concern in daily clinical practice [5,6]. Radiosensitization that could work synergistically with local irradiation both spatially and temporally therefore warrants further exploration.

Over the past decade, numerous research studies have focused on combining gold nanoparticles (AuNPs) with RT [2,7,8]. Due to their unique physiochemical properties, AuNPs have been widely used as a radiosensitizer in research fields. Promising results in in vitro and in vivo applications have also been reported [2,7,8,9,10,11]. AuNPs are biocompatible and can be designed in specific sizes or shapes (e.g., spheres, cubes, rods, cones, and other 3D structures) based on the delivery requirements for specific tumors. Moreover, the leaky vasculature with compromising lymphatic drainage, referred to as the enhanced permeability and retention (EPR) effect, allows AuNPs (typically sized 1–100 nm) into the tumor tissue [8,12]. These advantages make AuNPs a radiosensitizer that is superior to conventional macro-sensitizers [10]. The main mechanism supporting radiosensitization is the enhancement of the photoelectrical phenomenon during irradiation, since the mass energy absorption coefficient of high-Z AuNP is much higher than that of low-Z soft tissue. The subsequent emissions of secondary electrons will deposit doses in localized regions and greatly increase damage to DNA double strands.

The increased energy deposition with enhanced photoelectric effects in cancer cells is most prominent for kilovoltage (KV) RT. This inherently exhibits low penetration, which is not suitable to treat deep-seated lesions. In contrast to KV X-rays, the use of external beam of megavoltage (MV) X-rays is essential to achieve skin sparing and adequate dose deposition to deep-seated or large tumors. There have been encouraging demonstrations of AuNP-mediated radiosensitization when applying clinically suitable MV energies. Nevertheless, those promising results rely on high systemic concentrations of AuNPs [13,14,15], or the further modification of AuNPs with targeting moieties such as thioglucose [16,17], HER-2 (human epidermal growth factor receptor-2) [18], or goserelin [19] depending on specific tumor types. Investigating AuNP-mediated radiosensitization using clinical-utilized MV X-rays, with a clinically non-prohibitive amount of gold, and with a schedule approach similar to clinical practice is warranted.

Sonoporation is the use of sound—typically at ultrasonic frequencies—to temporarily modify the permeability of the cell plasma membrane, and this has been exploited to enhance the intercellular delivery of therapeutic materials and genes [20]. The key to effective sonoporation is the induction of cavitation, which is the process of microbubble (MB) formation, resonance, and destruction, during which the destruction of MBs in an acoustic field would disrupt both the endothelial wall and tumor cell membrane. It has been successfully demonstrated that combining MBs with ultrasound can greatly enhance the delivery efficiency in gene therapy [20] and AuNPs [21,22]. Our previous studies have demonstrated the enhanced delivery of AuNPs via the use of AuNP-encapsulated MBs (AuMBs) as a contrast agent in plasmonic photothermal therapy (PPTT) [22,23]. Moreover, the sonoporation rate was found to be linearly correlated with the inertial cavitation dose (ICD), which was measured from the corresponding spectral broadband signal during ultrasound stimulation [20,24]. Applying focused ultrasound locally increased the uptake of AuNPs at the target lesion. The approach of stimuli-responsive AuNP delivery offers an appealing strategy for administering a radiosensitizer.

Ultrasound has always been an important imaging tool in the diagnosis and treatment of liver cancer. By combining MBs as the contrast agent, ultrasound could increase the accuracy of liver cancer staging [25,26,27]. The present study assessed the effects of radiosensitization on human hepatocellular carcinoma, for which the radiotherapeutic effects are typically unsatisfactory [28]. We hypothesize that the augmented delivery of AuNPs through sonoporation with AuMBs to human hepatocellular carcinomas promotes the subsequent radiosensitization when applying MV X-rays.

## 2. Methods and Materials

### 2.1. Hepatocellular Carcinoma Cell Lines

The human hepatocellular carcinoma cell line Huh7 was obtained from the JCRB cell bank (Okayama, Japan). Cells were cultured in DMEM, supplemented with 10% fetal bovine serum and 50 U/mL penicillin/streptomycin, and kept at 37 °C in a humidified atmosphere of 5% CO_2_ and 95% air. For in vitro experiments, the Huh7 cells were trypsinized off and made into a suspension with 2 × 10^6^ cells/mL in each arm of the experiments.

### 2.2. AuNPs

The AuNPs were synthesized by the electrochemical conversion of an anodic gold material into particles using an electrolytic cosurfactant system; the procedure was developed previously and has been reported elsewhere [24]. To enhance the in vivo stability and avoid nonspecific cellular uptake by the reticular endothelial system, polyethylene glycol (PEG) AuNPs were synthesized. The procedure involved an EDC (1-ethyl-3-[3-dimethylaminopropyl] carbodiimide)-mediated coupling reaction and subsequent attachment of a blocking agent (mPEG-SH, which is a thiol-terminated methoxypoly [ethylene glycol]) at nonspecific adsorption sites on the AuNPs. Transmission electron microscopy (TEM, H7100, Hitachi, Tokyo, Japan) and the optical density (OD) of the AuNPs was investigated using spectrophotometry (at a dilution ratio of 1/100) to confirm the distribution and the optical properties of the AuNPs. The concentration of AuNPs was adjusted to 30 nM (molar concentration of AuNPs, equivalent to weight concentration of 1.1 mg/mL of gold) for each batch used in this study.

### 2.3. AuMBs

AuMBs were produced using the following emulsion method: a sonicator (Model 102C, Branson, Danbury, CT, USA) was used to sonicate a premixed solution—which contained 4% (*w*/*v*) HSA (human serum albumin, Octapharma, Vienna, Austria), 6 nM AuNPs, phosphate-buffered saline (PBS), and C_3_F_8_ gas—for 3 min followed by resting on ice for 5 min. Centrifugation was then performed at 1100 rpm for 3 min. The concentration and size distribution of the AuMBs were measured by a cell counter/analyzer using a 30-µm aperture (Coulter MultiSizer III, Beckman Coulter, Brea, CA, USA). AuMBs with a mean size of 1–3 µm were selected by varying the centrifugation parameters.

### 2.4. In Vitro Sonoporation System Setup

**Figure 1:**
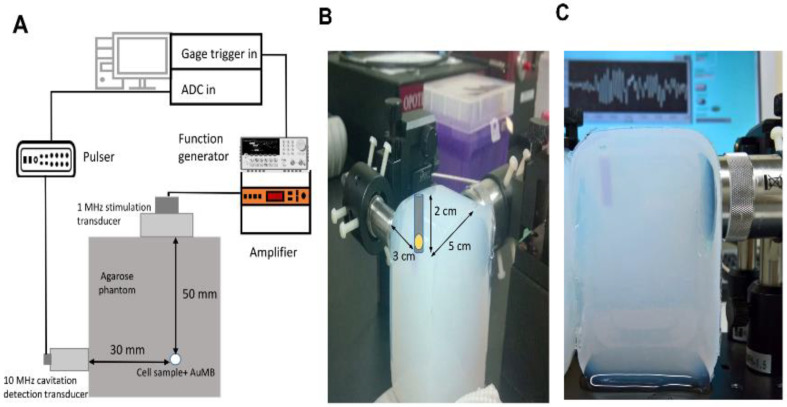
System setup for the ultrasound treatment and cavitation measurements. (**A**) Schematic of the in vitro treatment and inertial cavitation dose (ICD) measurement system. (**B**) and (**C**) Water-equivalent agarose phantom with a prespecified cell well at the focuses of two ultrasound transducers.

The system setup is shown in Figure 1. A cylindrical cavity with a diameter of 5 mm and a depth of 2 cm was made in an agarose phantom (2% concentration) as the container for a mixture of 200 µL of Huh7 cells (4 × 10^5^ cells) and 300 µL of AuMBs (2.6 × 10^8^ AuMBs). The corresponding concentration of AuNPs in the Huh7/AuMBs mixture was 3.6 nM, with a weight concentration of gold approximately around 135.6 µg/mL.

A 1-MHz cylindrical focused transducer (V303, Panametrics-NDT, Waltham, MA, USA) was applied to induce cavitation, and an unfocused 10-MHz transducer (V312, Panametrics-NDT) was used to receive signals from the AuMBs during ultrasound stimulation. The two transducers were positioned perpendicular to each other such that the –6-dB focal zone of the 1-MHz transducer covered the entire cylindrical cavity. The total sonoporation time was 5 min, with a pulse repetition frequency (PRF) of 100 Hz and a peak negative pressure of 0.568 MPa. The pulse duration was 30 µs in the long-duration ultrasound arm (L_US) and 10 µs in the short-duration ultrasound arm (S_US).

### 2.5. Cavitation Measurements

The inertial cavitation was measured as the ICD. It was quantified as the root-mean-square (RMS) value of the spectrum between 9.5 and 10.5 MHz, received by the 10-MHz transducer. Finally, the background (the RMS amplitudes measured for PBS alone) was subtracted, and the resulting amplitude was referred to as the differential ICD (dICD) [20]. The dICD was calculated as the average over 2 s of stimulation (200 pulses).

### 2.6. Assessment of Intracellular AuNPs

The intracellular concentration of AuNPs was assessed using inductively coupled mass spectrometry (ICP-MS). The cell solution was sonoporated and then centrifuged for 1 min at 1000 rpm, and the obtained pellet was washed to remove extracellular AuNPs. After washing, the pellet was dissolved with 2-mL aqua regia at 75–85 °C for 4 h. The solution was serially diluted to 10 mL with 2% HCl and then quantified by an ICP-MS (Agilent 7700e, Santa Clara, CA, USA). A series of gold standard solutions (0, 4, 8, 12, 16, and 20 ppb) were prepared with 2% HCl before each experiment. The resulting calibration curve was used to determine the intracellular amount of gold.

### 2.7. Colony Formation Assay

The Huh7 cell suspensions were diluted and seeded in six-well plates (Corning, Somerville, MA, USA) with appropriate number of cells after sonoporation (1250 cells/well for 0, 2, and 4 Gy; 2500, 5000, 7500 cells per well for 6, 8, and 10 Gy irradiation, respectively). After 2 h of incubation, the plates were irradiated at different doses (from 0 to 10 Gy) using a 6-MV photon linear accelerator (Elekta, Stockholm, Sweden). The irradiated cells were surrounded by a water-equivalent bolus that could bring scattered photons of lower energy in order to maximize the AuNP-induced effects [9] (Supplementary Figure S1). The cells were then cultured for 10 days. The number of colonies in each well (in clusters with >50 cells) was manually counted using an inverted phase-contrast microscope at 100X magnification after staining with cresyl violet.

### 2.8. γ-H2AX Immunofluorescence Microscopy

After irradiation at 4 Gy, the cells were incubated for 1 h, washed with PBS, fixed in 4% formaldehyde/PBS for 30 min, permeabilized in 0.5% Triton X-100 in PBS for 1 h, blocked in 5% bovine serum albumin for 1 h at room temperature, incubated with the antibody (FITC [fluorescein isothiocyanate]-conjugated antiphosphohistone γ-H2AX [Ser139], 1:1500; GeneTex, Irvine, CA, USA) for 2 h at room temperature in the dark, washed with PBS, and mounted in Vectashield mounting medium containing diamidino-2-phenylindole (Vector Laboratories, Burlingame, CA, USA). The γ-H2AX foci were counted per nucleus in each sample using a fluorescence microscope (Axio Imager A1, Zeiss, Oberkochen, Germany) at high magnification. The average number of γ-H2AX foci was calculated for at least 50 nuclei.

### 2.9. Western Blot Analysis

After being irradiated, the cells were incubated for 24 h and then lysed with protease inhibitor. Aliquots of cell lysates were loaded at adequate amounts onto gels and then subjected to electrophoresis. Samples was then transferred to a polyvinylidene fluoride membrane (PVDF, Merck Millipore, Burlington, MA, USA) and further incubated with primary antibodies to phosphorylated ATM (pATM, GeneTex, Irvine, CA, USA), poly (ADP-ribose) polymerase-1 (PARP1, Cell Signaling Technology, Danvers, MA, USA), γ-H2AX (GeneTex), and β-tubulin (Merck Millipore, Burlington, MA, USA). Bound antibodies were detected using appropriate peroxidase-coupled secondary antibodies followed by enhanced chemiluminescence (ECL, Boehringer Mannheim, Mannheim, Germany). Images were obtained after washing out the secondary antibody. The fluorescence images were visually scanned for luminescence and quantitatively analyzed using ImageJ software.

### 2.10. Targeted AuMBs with VEGFR2 Conjugation

To enhance the delivery of AuNPs in mice, we further modified the AuMBs with vascular endothelial growth factor receptor 2 (VEGFR2) as the tumor-specific target. VEGFR2 has been reported to be overexpressed on the endothelium of blood vessels in Huh7 tumors [29,30]. An avidin–biotin interaction was applied to provide noncovalent coupling of anti-VEGFR2 antibodies (anti-FLK1, Avas 12a1; eBioscience, San Diego, CA, USA) onto the AuMB shells. The method used to produce the VEGFR2-targeted AuMBs is presented in the Supplementary Materials (Figures S2 and S3).

### 2.11. Targeted AuMB-Enhanced Ultrasound Imaging in Mice

BALB/cAnN.Cg-Foxn1nu/CrlNarl mice (5 weeks of age) were obtained from the National Laboratory Animal Center and used for ectopic (subcutaneous) xenograft implantation. The in vivo experimental protocol was approved by the Institutional Animal Care and Use Committee of National Taiwan University (approval number IACUC 20160323). A 100-μL aliquot containing HuH7 cells (2.5 × 10^6^) in 1:1 PBS/Matrigel solution was injected subcutaneously into the right thigh of the mice. Ectopic tumors were allowed to reach a size with a longest axis of 6–8 mm before performing the subsequent experiments. The mice were kept in left lateral decubitus on a heated platform to maintain their body temperature during anesthesia. One hundred and fifty microliters of VEGFR2-targeted AuMBs or nontargeted AuMBs were administered in 15 s via a retro-orbital injection [31]. Real-time ultrasound imaging of the tumor was performed using a dedicated small-animal high-resolution unit (Prospect 3.0, S-Sharp, New Taipei, Taiwan) with a 20-MHz transducer for harmonic imaging.

### 2.12. AuMB-Sonoporation-Mediated Radiosensitization

The ectopic tumors in all mice were allowed to reach a size with a longest axis of 6–8 mm before receiving an injection (1) of normal saline, (2) normal saline plus RT, (3) VEGR2-targeted AuMBs with ultrasound sonoporation, or (4) VEGFR2-targeted AuMBs with ultrasound sonoporation plus RT. One hundred and fifty microliters of normal saline or VEGFR2-targeted AuMBs was administered via a retro-orbital injection, and 90 s later a 1-MHz focused, 200-μs-long ultrasonic pulse with a pulse repetition rate of 200 Hz and peak negative pressure of 0.838 MPa was administered. The sonoporation pulses were applied for 30 s, followed by a 10-s rest period to allow the blood vessels to refill with MBs. After five courses of sonoporation, another 150 microliters of AuMBs was administered followed by the same sonoporation protocol. The total sonoporation time was 5 min. The intravenous administration of gold was approximately 122 µg within 3 days, which was about 4.7 μg of gold for every gram of mice weight.

Two hours after the AuMB sonoporation, each mouse was immobilized using a customized harness. With its body shielded, the tumor on the right thigh was irradiated with a half-beam rectangular field of 20 × 10 cm using a 6-MV photon linear accelerator at a dose rate of 440 monitor unit/second (Supplementary Figure S4). The monitor units set for irradiation dose in were calculated by a national-qualified physicist and verified with an ion chamber. The radiotherapy comprised three fractions of irradiation at 10 Gy/day consecutively, with the mice receiving corresponding pre-RT treatment. The tumor volume was calculated as (a × b^2^)/2, where a and b are the length and width of the tumor, respectively. The tumor volume was measured every 3 days until the 34th day after irradiation. Humane endpoints were determined according to a clinical scoring system developed by the Institutional Animal Care and Use Committee in our institution.

### 2.13. Statistical Analysis

All sets of analyzed statistical data were obtained from three independent experiments. Statistical significance between two experimental groups was analyzed with the two-tailed unpaired Student’s *t* test. The significance criterion was set as *p* < 0.05. The analyses were performed using the MedCalc statistical software (version 19.1.3, MedCalc Software, Ostend, Belgium).

## 3. Results

### 3.1. Characterization of AuNPs and AuMBs

**Figure 2:**
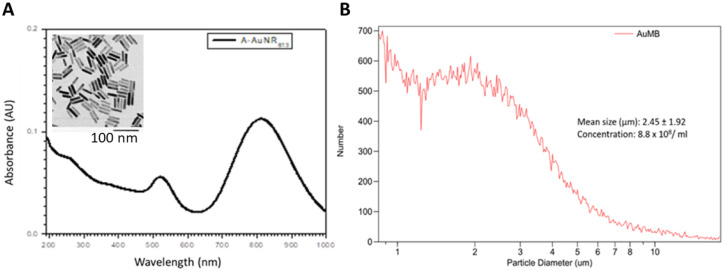
Characterization of god nanoparticles (AuNPs) and AuNP-encapsulated microbubbles (AuMBs). (**A**) The optical absorption peaked at 813 nm. The inset transmission electron microscopy image accurately shows the aspect ratio of the rod-shaped AuNPs. (**B**) Size distribution of the AuMBs.

The AuNPs were well dispersed in aqueous solutions and typically had a rod-like shape with a 1:45 aspect ratio by TEM (10.3 ± 2.8 nm in diameter and 46.1 ± 7.7 nm long, mean ± SD). Transmission electron microscopy and spectrophotometry were used to assess the geometric characteristics and OD of the AuNPs (Figure 2). The optical absorption peaked at 813 nm. Figure 2 also shows the size distribution of the AuMBs; they had a size of 2.45 ± 1.92 µm (mean ± SD) and their concentration was 8.8 × 10^8^/mL.

### 3.2. Acoustic Cavitation Effect and Intracellular Localization of AuNPs

**Figure 3:**
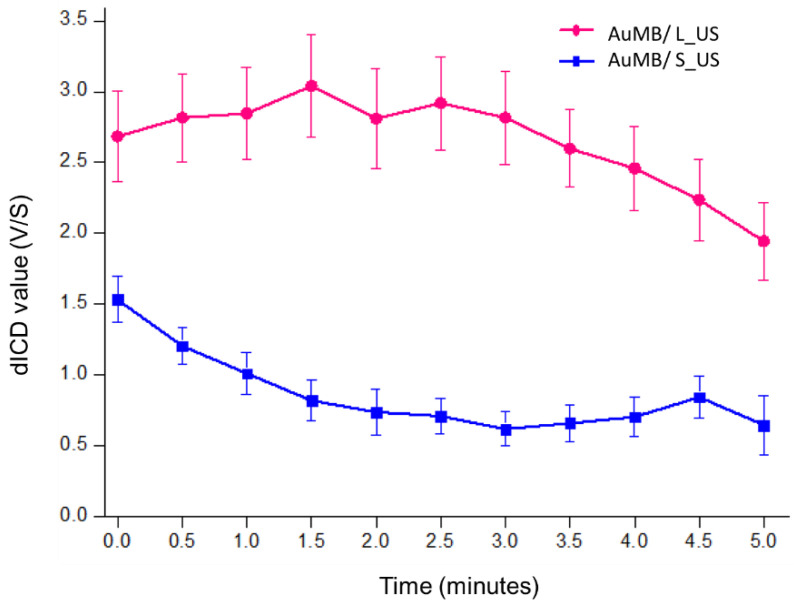
Differential inertial cavitation dose (dICD) values measured during 5 min of sonoporation with long-duration (pulse duration = 30 µs) and short-duration (pulse duration = 10 µs) acoustic stimulation. Data are mean and SD values.

The dICD was measured and calculated every 30 s during the 5 min of ultrasound stimulation. Figure 3 demonstrates that the dICD was significantly higher for a longer pulse duration of ultrasound (L_US vs. S_US)**.**

**Figure 4:**
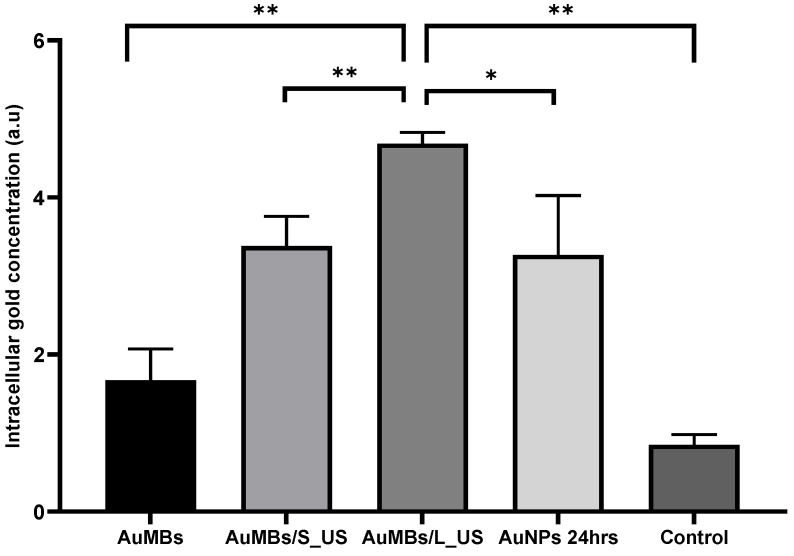
Results of a quantitative analysis of the intracellular uptake of AuNPs by ICP-MS. Long-duration acoustic sonoporation (pulse duration = 30 µs) was associated with a significantly higher intracellular concentration of AuNPs compared to short-duration (pulse duration = 10 µs) acoustic stimulation and negative controls. Data are mean and SD values from three independent experiments. * *p* < 0.05, ** *p* < 0.005.

To assess the delivery efficiency of AuNPs by sonoporation, we additionally incubated the Huh7 cells with 3.6 nM AuNPs (the same concentration used in the sonoporation experiments) in a regular medium for 24 h as a comparative arm. As shown in Figure 4, ICP-MS quantification demonstrated that the intracellular concentration of AuNPs was increased by AuMB sonoporation. Most importantly, the intracellular AuNP concentration was significantly higher for sonoporation at a longer pulse duration (4.7 vs. 3.3 arbitrary units [a.u.], *p* = 0.005). Moreover, the intracellular concentration of gold after 5 min of sonoporation was comparable (if not higher than) that after 24 h of incubation with AuNPs (4.7 vs. 3.4 a.u., *p* = 0.03).

### 3.3. Clonogenic Assessment of In Vitro Radiosensitization by AuMB Sonoporation

**Figure 5:**
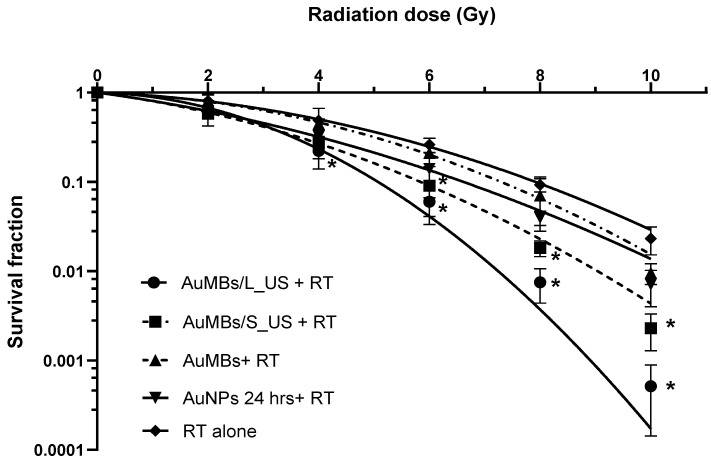
The numbers of colonies in each well containing more than 50 cells were counted as the quantitative results of the clonogenic assays. Data are mean and SD values from three independent experiments. * *p* < 0.05 compared with RT alone.

Figure 5 shows that irradiation decreased the Huh7 cell survival in a dose-dependent manner. Five minutes of AuMB sonoporation significantly enhanced the radiation-induced reduction in cell survival. With RT at more than 6 Gy, applying pre-RT treatment with AuMB sonoporation led to significant radiosensitization for ultrasound stimulation with either a long pulse duration (L_US) or a short pulse duration (S_US). For irradiation at 4 Gy, only AuMBs with L_US sonoporation achieved significant radiosensitization. In contrast, no radiosensitization was observed in groups of cells treated with AuMBs alone and radiation. The clonogenic surviving fractions were fitted to a linear-quadratic (LQ) model: fraction of cells surviving = *e*^–(*A* × *D* + *B* × *D*^2)^ [32]. The statistical coefficients (*R*^2^ values) for fitting the LQ model to the clonogenic curves were >0.997 for all arms of the experiments. We then derived the dose-modifying ratio (DMR) for quantifying the radiosensitizing power of AuMB sonoporation as follows:

$$\;\mathrm{DMR}x=\frac{Dose\;without\;AuNPs}{Dose\;with\;AuNPs}\;(\mathrm{for}\;x\%\;\mathrm{survival}).$$

In our colony formation assay, we obtained a DMR_10%_ of 1.5 ± 0.45 for AuMBs/L_US, 1.32 ± 0.28 for AuMBs/S_US, and 1.19± 0.19 for the 24-h-incubation arm at a surviving fraction of 10%.

### 3.4. γ-H2AX Immunofluorescence Staining

**Figure 6:**
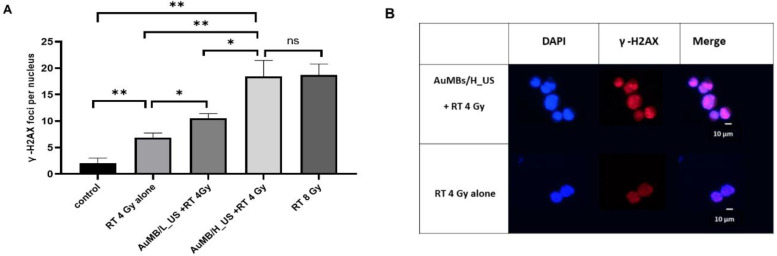
(**A**) The number of γ-H2AX foci were counted in 50 cells at 1 h after the treatment. Data are mean and SD values for the number of foci per cell in each group from three independent experiments. * *p* < 0.05, ** *p* < 0.005. (**B**) Representative graphs of Huh-7 cells at 1 h after the treatment of radiotherapy (RT) with or without preceding AuMB-sonoporation. Cells were stained with γ -H2AX and 4’,6-diamidino-2-phenylindole (DAPI).

Double-stranded DNA breaks were assessed by calculating the number of γ-H2AX foci at 1 h after irradiation at 4 Gy. Irradiation at 8 Gy served as a positive control. Figure 6 demonstrates that pre-RT treatment with L_US (18.4 vs. 6.8 foci) or S_US (10.5 vs. 6.8 foci) AuMB sonoporation produced significantly more immunofluorescent foci compared to RT at 4 Gy alone. Without ultrasound-mediated sonoporation, there was no enhancement in DNA damage by applying pre-RT treatment with AuMB immersion alone, compared with RT alone (7.2 vs. 6.8 foci, *p* = 0.67). Consistent with our findings from the ICP-MS quantification of intracellular AuNPs, the γ-H2AX foci increased with ultrasound stimulation in a dose-dependent manner. Notably, the number of foci for L_US was comparable with that in the arm with double-dose irradiation (8 Gy).

### 3.5. Western Blotting

**Figure 7:**
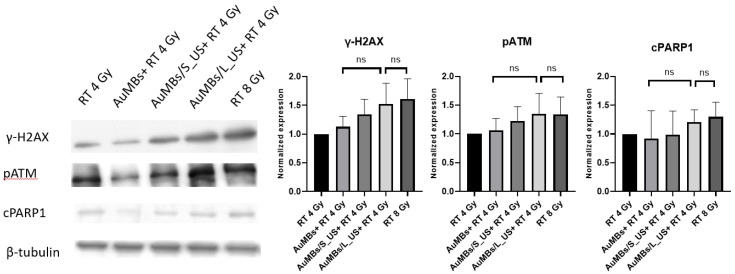
Representative Western blots and densitometric analyses of the levels of the DNA damage indicators (pATM and γ-H2AX) and the apoptosis marker cleaved PARP1 (cPARP1). *n* = 3 in each treatment arm.

As shown in Figure 7, Western blotting revealed that pre-RT treatment with AuMB sonoporation enhanced the expression of DNA damage indicators (pATM and γ-H2AX). Moreover, the level of the apoptosis marker (cleaved PARP1) also increased after pre-RT treatment with AuMB sonoporation. In contrast, pre-RT AuMB immersion without ultrasound stimulation was not associated with an elevated expression of the targeted markers compared with RT alone. Although the enhanced expressions of the prespecified markers did not reach statistical significance, the trend of an ultrasound-dose-dependent increase echoed our findings from the intracellular assessments of AuNPs (Figure 4), clonogenic assay (Figure 5), and γ-H2AX immunofluorescence staining (Figure 6).

### 3.6. VEGFR2-Targed AuMBs to Enhance In Vivo Delivery

**Figure 8:**
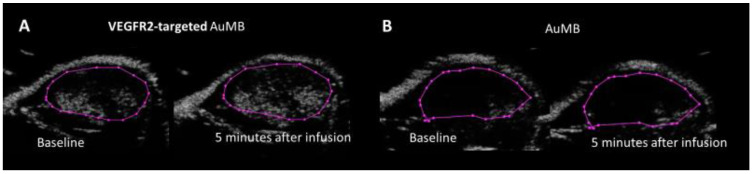
Representative harmonic B-mode ultrasonic images (20-MHz transducer) of xenograft tumors with VEGFR2-targeted AuMBs (**A**) or AuMBs (**B**) infused at baseline and 5 min later. The dynamic range was 50 dB with gain of −26 dB. The average enhancement in the pixel intensity was 7.84 vs. 0.27 a.u. (*p* < 0.05).

The VEGFR2-targeted AuMBs had a size of 2.42 ± 1.85 μm and a concentration of 9.1 × 10^8^/mL, which did not differ significantly from nontargeted-AuMBs. After confirmation by flow cytometry and a binding assay (Supplementary Materials), VEGFR2-targeted AuMBs were utilized in further in vivo experiments. Figure 8 shows the harmonic B-mode images obtained using a 20-MHz transducer. The average enhancement in the pixel intensity was significantly higher for VEGFR2-targeted AuMBs infusion (Figure 8A) than for nontargeted AuMBs (7.84 vs. 0.27 a.u., *p* < 0.05) (Figure 8B).

### 3.7. In Vivo Assessment of Xenograft Tumor-Growth Delay

**Figure 9:**
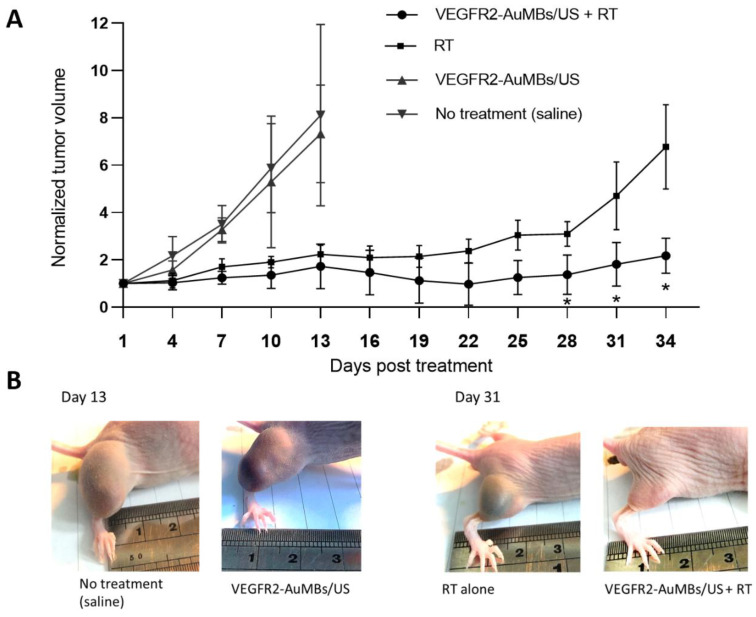
Tumor growth in Huh7 xenograft models with and without AuMBs/sonoporation prior to 6-MV irradiation. (**A**) Normalized tumor volume after treatment (*n* = 3 in each group). (**B**) Representative mice from each group. * *p* < 0.05, compared with RT alone. Data are mean and SD values.

As shown in Figure 9, while tumor growth delay was achieved by RT alone at 30 Gy in three fractions, treatment with AuMB sonoporation alone did not delay tumor regrowth compared to the control group (normal saline infusion). Combined treatment with AuMB sonoporation and RT significantly delayed the regrowth of the established subcutaneous Huh7 ectopic tumors compared with both RT alone and the control group (*p* < 0.05 at posttreatment days 28, 31, and 34).

## 4. Discussion

We have successfully demonstrated MV radiosensitization in human liver carcinoma, both in vitro and in vivo, using pre-RT treatment with AuMB sonoporation. The induction of cavitation-assisted sonoporation significantly increased the intracellular concentration of AuNPs, along with more DNA damage and ultimately cell death. While the AuNP-mediated radiosensitization is greatly affected by the subjected cell lines, and the geometric condition (size/shape) of AuNP, but also by the condition of irradiation (dose/dose rate, beam characteristics) [11,33], we have confirmed the radiosensitization power of our AuNPs by incubation for 24 h. The clonogenic assay (Figure 5) revealed a modest DMR of 1.19. The enhanced delivery of AuNPs by sonoporation was associated with a higher DMR of 1.56, which is consistent with previous findings for surface-modifying AuNPs and MV irradiation [19]. The total amount of gold used in vivo was comparably low (less than 5 µg of gold for per gram of mice weight). In contrast to previous studies that required 24 h of incubation with AuNP in vitro [16,17,18,19] or the infusion of a large amount of gold in vivo (approximately 1~2 mg of gold per gram of body weight) [14,15], our strategy represents a step forward for facilitating preclinical translation. To our knowledge, this study has provided the first demonstration of the sonoporation-assisted delivery of AuNPs for MV radiosensitization. Several previous reviews have addressed the mechanisms of AuNP-induced radiosensitization. Explorations on how intracellular AuNPs interact with cellular components besides DNA—the primary biological target of irradiation—were beyond the scope of this study.

The principle underlying sonoporation-mediated delivery is the induction of reversible pores on the cell membrane. The diameters of these pores range from 100 nm to several micrometers, allowing normally impermeable extracellular agents to enter the cell cytoplasm [34,35,36]. We have previously assessed the correlation between the dICD and the sonoporation rate of short-chain DNA [20]. In the present study, we adopted settings and acoustic parameters for in vitro sonoporation that were similar to those in our previous studies [23]. A linear correlation between the number of acoustic cycles and the efficiency of delivering AuNPs was demonstrated in our previous work [22]. Therefore, pulse durations of 30 and 10 µs were chosen as the experimental factor for AuNP sonoporation. Short ultrasound exposure times (on timescales from microseconds to milliseconds) may be sufficient to induce intracellular uptake in experiments where the cells are attached to the upper surface of a small chamber, within which the MBs are in close vicinity to the cells. In contrast, in our in vitro experiments, the Huh7 cells were loaded freely floating into an agarose well. We applied long durations of ultrasound stimulation (with a total exposure time of 5 min and average duty cycles of 0.1 to 0.3%) to allow interactions to occur between AuMBs and cells, which was similar to the conditions used in previous investigations using cell suspensions with sonoporation [37,38].

Several studies have demonstrated that combining low-intensity focused ultrasound with MBs is an effective radiosensitization method. This approach has significantly reduced the radiation dose required for killing tumors in prostate cancer [39], fibrosarcoma [40], and bladder cancer [41]. In those previous studies, after injecting MBs, xenograft tumors were exposed to a 16-cycle, 50-ms tone burst with a center frequency of 500 kHz and a PRF of 3 kHz. The treatments lasted for 5 min and the ultrasound peak negative pressure was 0.570 MPa [39,40,41]. Together with MBs, the ultrasound-induced vascular damage greatly enhanced the radiation effect, making a daily radiation dose of 2 Gy as effective as an ablative dose of 8 Gy [39]. Ceramide-related endothelial cell apoptosis leading to vascular disruption has been proposed as the mechanism underlying radiosensitization. Although some investigations have claimed that vascular disruption played a role during high-dose irradiation, the actual contributions from endothelial cells to the tumor response to radiation remain controversial [42,43]. Besides, a PRF as high as 3 kHz is not usual in research on ultrasound-guided drug delivery involving animal models [44,45,46,47], in which PRFs ranging from 1 to 100 Hz have been reported most frequently. In the present study, we combined a much longer pulse duration of 200 μs with a conservative PRF of 200 Hz (compared to a pulse duration of 30 μs with a PRF of 100 Hz in vitro) for a mouse model, based on the rationale of the presence of greater attenuation and the lack of immediate MB–cell contact. This approach is similar to that used previously in a study addressing gene delivery in mice [44].

Applying ultrasound irradiation to metal nanoparticles without MBs has recently been applied as a novel approach for cancer treatment, which is referred to as sonodynamic therapy [48]. A very recent study demonstrated the occurrence of radiosensitization with ultrasound stimulation and gold nanospheres after X-ray irradiation in cervical cancer cells [49]. The therapeutic efficacy can mostly be attributed to the generation of reactive oxygen species and the mechanical cytotoxic effects of cavitation, although the exact underlying mechanism remains unclear [48]. Notably in our works, while the intracellular concentration of gold was comparable between cells treated by 24-h immersion of AuNPs and AuMBs/S_US (Figure 4), the sonoporation arm had a worse survival following irradiation (Figure 5). Enhanced radiation response with ultrasound-stimulated MBs in vivo has been reported and a disruption of endothelial cell is attributed for the mechanism (discussed above). However, it is interesting to observe the effect in cell line experiments. We think that the generation of reactive oxygen species and the mechanical cytotoxic effects of cavitation, the principle of sonodynamic therapy, is a reasonable explanation. Further investigation on how cavitation affects radiosensitivity, especially beyond the impact on endothelium is warranted. On the other hand, cells immersed in AuMB without ultrasound achieved a modest, if any, radiosensitization. They therefore had a similar survival to cells treated by 24-h immersion of AuNPs, given with a significantly lower intracellular concentration of gold (Figure 4). We think the effect could probably be attributed to the higher peri-cellular concentration of AuNPs under the setting of AuMBs to cell ratio of 650:1. AuNPs aggregating on the plentiful AuMBs were more likely to attached to the cells, compared to those randomly dispersed in medium for 24-h incubation. These increased the interactions between AuNPs and ionization.

To enhance the tumor-specific delivery of AuMBs in live animals, we used VEGFR2 as the molecular target. VEGFR2 is primarily localized to—and significantly up-regulated on—the tumor vasculatures in various human solid cancers [29,41]. VEGFR2-targeted MBs have been used for ultrasound imaging in mouse models of a wide range of tumor types, including colon cancer [50,51], liver cancer [52], bladder cancer [53], breast cancer [54], and melanoma [55]. It has been shown that an acoustic radiation force can push targeted MBs toward the vessel wall during ultrasound molecular imaging and thereby promote the binding and retention of MBs in the diseased area [35,56]. Furthermore, ultrasound-induced cavitation, by increasing vessel permeability and generating convective flow, will enhance the extravasation of biochemical agents to the tumor [57]. In research using focused ultrasound with drug-loaded microbubbles for rat tumors, additional VEGFR2-targeting have been demonstrated significantly enhance drug release and reduce tumor progression in a rat model [58]. We have also previously used VEGFR2-targeted AuMBs to enhance the therapeutic ratio of PPTT in a mouse model [22]. In that study, nonlinear optical microscopy confirmed the increased AuNP delivery to tissues after acoustic stimulation and targeted injection of AuMBs. In the present study, harmonic ultrasound B-mode imaging confirmed the superiority of tumor-specific delivery of the VEGFR2-targeted AuMBs over the nontargeted AuMBs. We further demonstrated that combining VEGFR2-targeted AuMBs with sonoporation induced MV radiosensitization in human liver cancer cells. Further studies with orthotopic tumors are warranted to achieve the goal of theragnostic ultrasound for liver cancer treatment.

The present study performed a proof-of-concept investigation of spatiotemporally controllable AuNP-mediated radiosensitization with ultrasound. The strategy is unique in the following three critical points and thus is promising for realizing the therapeutic gain from AuNPs. First, these in vivo radiosensitization were demonstrated with an amount of gold that is considerably lower than that reported by literatures [13,14,19]. As toxicological studies to establish safe limits for clinical use of AuNPs are still pending, the low systemic dose of gold in our study offers an opportunity for the translation into clinics. Second, the mice in our study were irradiated with megavoltage X-ray in three fractionated daily doses of 10 Gy, a schedule more similar to clinical practice. By contrast, most of the in vivo studies evaluated the AuNP-mediated radiosensitization under one single high dose of irradiation (5–30 Gy) [2]. Besides, it has been reported that fractionated RT induced an accumulation of apoptosis proportional to the number of fractions of RT [59]. Thereby, the combination of AuNPs and fractionated irradiation may improve the therapeutic ratio [2]. Finally, VEGFR2, a target on the vasculatures of various solid tumors, was adopted to enhance the tumor-specific delivery of AuMBs. Compared with literatures using cancer-specific targets as the surface modifications on AuNPs [18,19], our approach seems more universal. In addition, the central frequency of ultrasound applied in the present work, was 1 MHZ, posing deep penetration in soft tissue. It could collaborate well with megavoltage RT targeting deep-seated tumor. The mechanical index of the ultrasound was about 0.9, which was much lower than the maximum threshold of 1.9 for clinical imaging [60]. Therefore, the promising results, in vitro and in vivo, make the strategy worthy of further translational investigations.

In summary, the present findings suggest that AuMBs—through cavitation-induced sonoporation—can effectively enhance the targeted delivery of AuNPs to cancer cells. Exposure to ionizing irradiation enhanced the DNA damage, thereby achieving radiosensitization with MV radiation. The enhanced delivery of AuNPs, DNA damage, and cancer-cell survival were all associated with the sonoporation in an ultrasound-dose-dependent manner. Although the detailed biological pathways and the interplay between the AuNPs and the cells remain to be fully elucidated, this study represents the first demonstration of MV radiosensitization in liver cancer cells through the sonoporation of AuNPs.

## Supplementary Materials

The following are available online at https://www.mdpi.com/1422-0067/21/21/8370/s1, Figure S1: Setup for in vitro irradiation, Figure S2: Flow cytometry of avidin-labeled VEGFR2-AuMBs, Figure S3: VEGFR2-AuMBs bonded to human umbilical endothelial cells (HUVEC), Figure S4: Setup for in vivo irradiation.
