# Supplementary material for: Enhanced Radiosensitization for Cancer Treatment with Gold Nanoparticles through Sonoporation
Source: Int J Mol Sci. 2020 Nov 8;21(21):8370. doi: 10.3390/ijms21218370 (PMC7664670; doi:10.3390/ijms21218370)

Supplementary Materials

Irradiation of cells

The radiotherapy of different doses of radiation was given by a Synergy Elekta 6-MV photon linear accelerator (Elekta AB, Inc, Stockholm, Sweden). The distance from the radiation source to the bottom of the flask was set at 100 cm. The cells were irradiated with different doses of radiation (0 Gy-10 Gy).

Supplementary Figure 1

The cell plate was irradiated by a 6-MV linear accelerator with a source-to-well distance of 100 cm. The cell plate was surrounded by the water-equivalent phantoms and bolus for full electronic equilibrium.

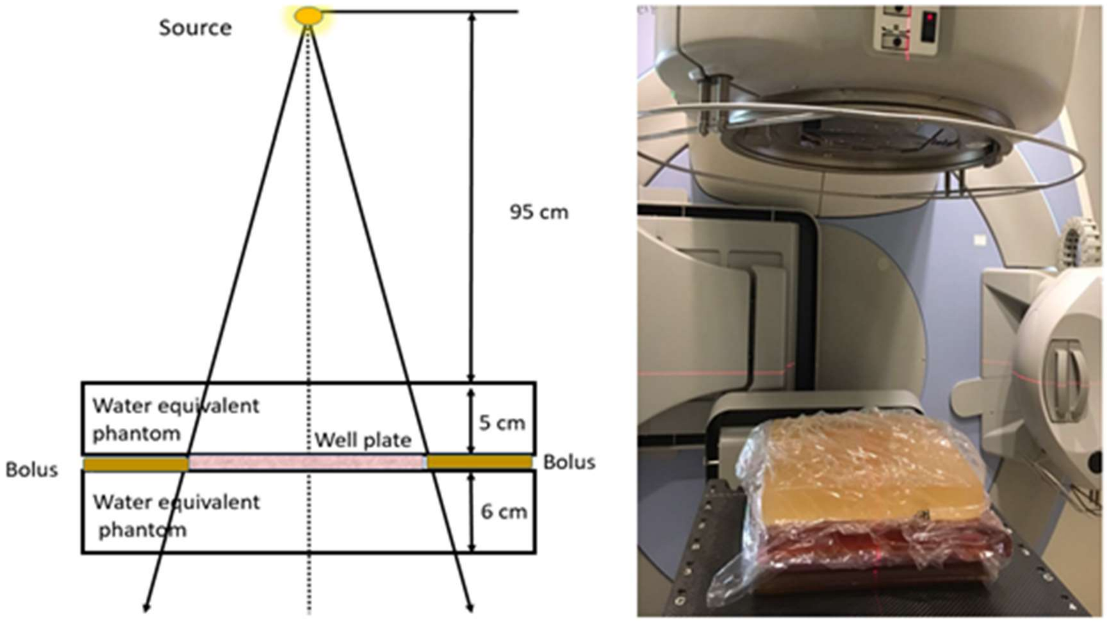

The production of flow cytometry and in vitro binding of VEGFR-targeted AuMB

We used avidin-biotin interaction to provide noncovalent coupling of anti-VEGFR2 antibodies onto the AuMB shells. Fluorescein (0.5%)-conjugated avidin (FITC-Av) was mixed with AuNP and HSA (1:1 volume ratio) at weight ratios (FITC-Av: HSA) of 1:50. Three volume part of PBS was then added into the mixture, followed by sonication with C_3F_8 gas. The setting of sonication was identical to that for untargeted AuMB. The Av-AuMB solution was then iced-rest and centrifuged. Before adding biotinylated rat monoclonal anti-VEGFR2 antibody (anti-FLK1, Avas 12a1; eBioscience, San Diego, CA, USA), the Av-AuMB was washed three times to remove free avidin and HSA. The amount of biotinylated of anti-VEGFR2 was 20 μl for 1 ml of Av-AuMB. Excess unbound antibodies were removed by saline washing. The anti-VEGR2-AuMB (later abbreviated as VEGR2-AuMB) was then analyzed by cell counter for size and number analysis. The VEGFR2- AuMB was produced by unlabeled avidin for further in vivo experiments. Flow cytometry (Supplementary Figure 2) and HUVEC binding assay (Supplementary Figure 3) were performed before animal studies. For flow cytometry, 50 μl of VEGFR2-AuMB were diluted in 1 mL of PBS and samples were analyzed for fluorescence using Becton Dickinson FACSCalibur™ system (Becton Dickinson, Franklin Lakes, NJ, USA). Data were analyzed using CellQuest software (Becton Dickinson). A total of 2000 events were captured for each sample in duplicate. As a control, the untargeted AuMB was also assessed as described above.

Supplementary Figure 2

Flow cytometry of avidin-labeled VEGFR2-AuMB (A) and untargeted AuMB (B). FITC-labeled avidin was successfully incorporated onto the shell of AuMB, making significant fluorescence compare to untargeted AuMB.

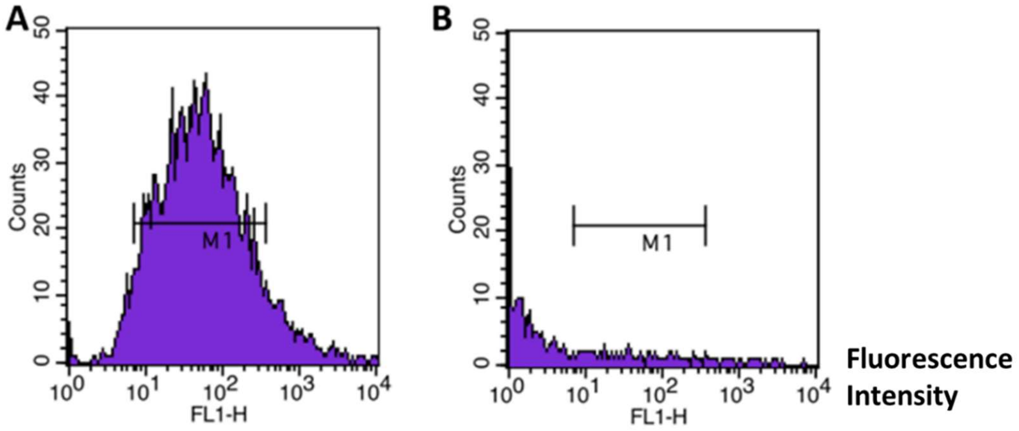

The human umbilical endothelial cells (HUVEC) that express high level of VEGFR2 were placed and grown on the glass slides. A total of 100 µl of VEGFR2-AuMB with FITC-labeled avidin were placed on an empty slide. A slide with HUVEC cells was placed on the top of the slide of the VEFGR2-AuMB. After 15 minutes of static exposure. The slide was washed 3 times to remove unbounded VEGFR2-AuMB. After binding of microbubbles, we then added Dylight 594-anti-VEGFR2 antibodies onto the slide to delineate the locations of VEGFR2. After removing excess Dylight 594 -anti-VEGFR2 antibodies, green fluorescence (FITC-avidin) and red fluorescence (Dylight 594- anti-VEGFR2) were detected using fluorescent microscopy. As a control, the untargeted AuMB was also assessed as described above.

Supplementary Figure 3

FITC-avidin labeled VEGFR2-AuMB bonded to human umbilical endothelial cells (HUVEC). FITC-fluorescence (green stain) mainly surrounded HUVEC cells. Dylight 594-anti-VEGFR2 (red stain) antibodies was then added onto the slide to delineate the locations of VEGFR2. The co-localization of green and red fluorescence implied the specific binding to VEGFR2-AuMB on the HUVEC through VEGFR2 binding.

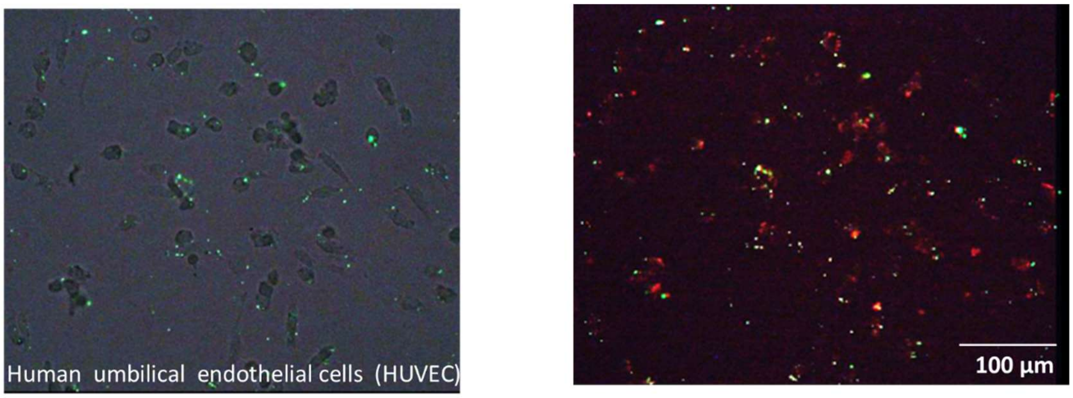

Supplementary Figure 4

Mice were immobilized using a customized harness. With the body shielded, the thigh tumor was irradiated with a half-beam rectangular field of 20×10 cm, at a source-to-tumor distance of 100 cm. A water-equivalent bolus was placed on the top of the harness to ensure full electronic equilibrium. A 6-MV photon linear accelerator was used to irradiate the thigh tumor with fractions of 10 Gy/day for 3 consecutive fractions.

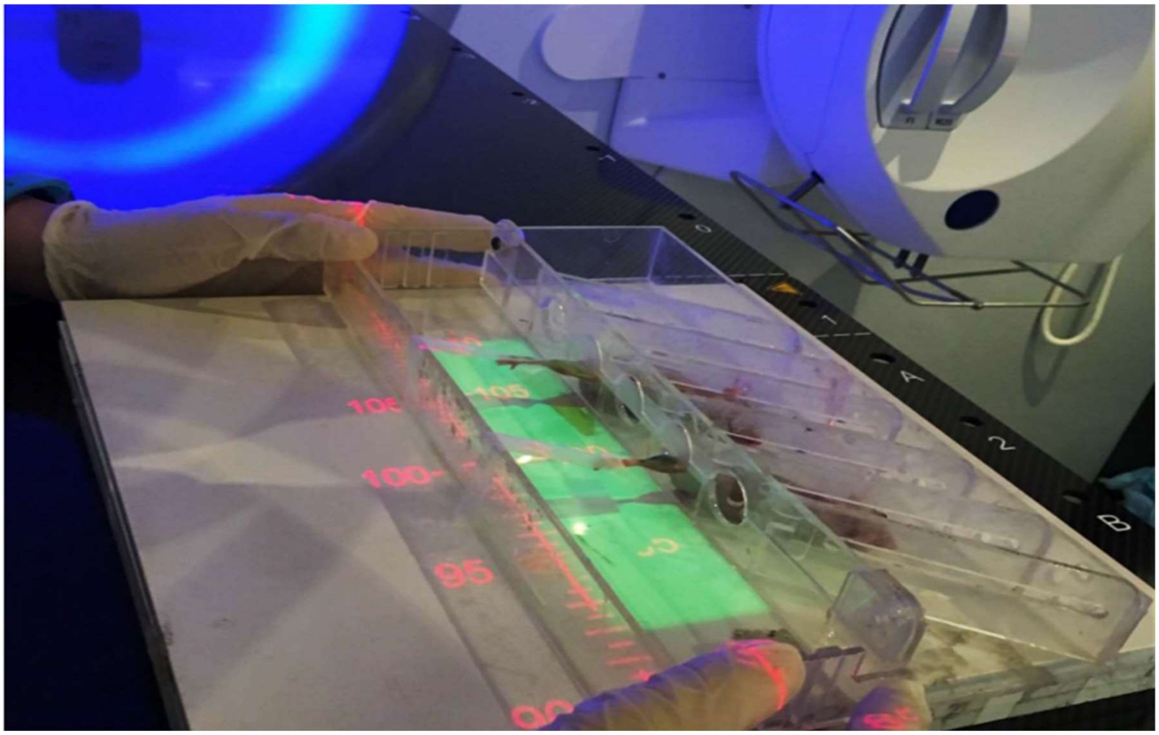

Supplement: Supplementary file 1 [file ijms-21-08370-s001.pdf]
